# Activities of national bioethics committees during the COVID-19 pandemic: publication trends and future challenges

**DOI:** 10.1186/s12910-026-01468-6

**Published:** 2026-05-13

**Authors:** Hiroto Takagi, Yusuke Inoue

**Affiliations:** Department of Healthcare Ethics, Kyoto University School of Public Health, Kyoto-shi, Kyoto, Japan

**Keywords:** COVID-19 Pandemic, Public Health, National Bioethics Committee, Transparency, Resource allocation, Public Health Emergency, Public Health Guidelines

## Abstract

**Introduction:**

National bioethics committees (NBCs) are expected to play a crucial role in addressing bioethical challenges. However, few studies have examined the activities of NBCs during the COVID-19 pandemic. This study investigated whether, and to what extent, NBCs were active during this public health emergency.

**Methods:**

Between June and November 2024, we compiled and analyzed statements, opinions, guidelines, and reports on the COVID-19 pandemic issued by NBCs in Organisation for Economic Co-operation and Development countries, the Council of Europe, and Singapore. We collected online publications related to COVID-19 from these committees, starting in 2019, and categorized them based on their publication date and themes. As the authors are proficient only in Japanese and English, publications in languages other than English were reviewed with the assistance of ChatGPT.

**Results:**

The study examined 43 NBCs established in 35 countries and one organization. Among them, 22 committees—based in 21 countries and one organization—published materials on ethical issues related to the COVID-19 pandemic. In total, these 22 NBCs issued 108 publications. We categorized the publications into 10 groups, with the three most frequently addressed topics being “vaccination (including vaccine distribution),” “frameworks for COVID-19 policies,” and “medical resource allocation.”

**Discussion:**

The results indicate that publications were issued promptly in response to the evolving crisis and addressed a broad spectrum of bioethical concerns. This study also identifies three challenges and areas for growth. First, when responding to urgent public health crises, NBCs must account for three key considerations: (1) the transparency of the statement publication process, (2) the incorporation of statements into public policies, and (3) protections for the persons involved in drafting such statements. Second, significant regional disparities exist in NBC activity levels. Finally, the findings underscore the need for a more coordinated and globally inclusive approach to bioethical governance in future public health emergencies.

## Introduction

According to the United Nations Educational, Scientific and Cultural Organization (UNESCO), “a ‘bioethics committee’ is a committee that systematically and continually addresses the ethical dimensions of (a) the health sciences, (b) the life sciences and (c) innovative health policies” to “benefit not only individuals [...] but entire societies and even the global community” [1]. To fulfill this purpose, national bioethics committees (NBCs) serve as “platforms and bodies for ethical debate, analysis and policy development” [1] and, hence, are also “expected to advise policy makers, politicians and law makers” [1] (see also [2, 3] for recent studies on the activities of NBCs).

The first NBC was the National Commission for the Protection of Human Subjects of Biomedical and Behavioral Research (1974–1978) in the United States, which was established under the 1974 National Research Act. While NBCs in the United States were not permanently established, the Comité consultatif national d’éthique (French National Advisory Ethics Council for Health and Life Sciences) was created as the first standing committee in 1983. Since then, NBCs have been set up in other European countries and, later, in other regions.

NBCs are expected to play a crucial role in addressing bioethical challenges. In many cases, NBCs focus on the ethical aspects of advanced medical technologies and biotechnologies as well as bioethical issues that do not fall under public health emergencies. Therefore, it is necessary to investigate how NBCs responded to the public health crisis posed by the COVID-19 pandemic. A previous study has examined the activities of the German Council of Ethics during the COVID-19 pandemic [4]. The present paper builds on that paper and expands its scope by offering an international comparison of NBCs in Organization for Economic Cooperation and Development (OECD) member countries. It also contributes to the broader field of global bioethics. As previous research has shown, NBCs play a significant role in fostering international dialogue [5]. Considering the global nature of pandemics like COVID-19, it is also crucial to explore whether NBCs contribute to cross-border cooperation through comparative analysis.

This examination is crucial for three key reasons. First, this study offers an opportunity to examine whether, and to what extent NBCs have accounted for the ethical principles articulated in the *Universal Declaration on Bioethics and Human Rights* [6]. This document, which calls for the establishment of NBCs (Article 19), outlines key ethical values such as the “respect for human vulnerability and personal integrity” (Article 8) and “equality, justice and equity” (Article 10) [6]. The justification of public health policies requires a careful balancing of the values these policies promote against those they may compromise, which is an approach commonly referred to as the proportionality principle [7]. This principle is particularly relevant in the context of pandemics. To meaningfully apply it, a prior awareness of the diverse ethical values at stake is essential. NBCs are expected to contribute to this awareness and deliberation by identifying and articulating these values.

Second, this article provides an opportunity to re-evaluate the importance of NBCs. Since the United States’ Presidential Commission for the Study of Bioethical Issues suspended its activities in 2016, the literature has paid relatively little attention to NBCs. The global nature of the COVID-19 pandemic offers a unique opportunity for a comparative analysis, allowing the exploration of similarities and differences in the approaches taken by NBCs across various countries.

Third, this study contributes to a broader reconsideration of the role of experts in (bio)ethics during emergencies. NBCs often focus on biomedical issues but lack substantial discourse on the ethical challenges that arise in crises such as pandemics. Examining their activities during the COVID-19 pandemic provides valuable insights into the roles experts should play in emergencies and helps to shape ethical frameworks for future crises.

## Methods

### Data sources

Between June and November 2024, we collected and classified statements, opinions, guidelines, and reports about the COVID-19 pandemic (henceforth, “publications”) published by NBCs in Organisation for Economic Co-operation and Development (OECD) countries. We also examined the publications of Singapore’s National Medical Ethics Committee and the Committee on Bioethics (DH-BIO) within the Council of Europe. These countries are generally considered to have stronger public health systems—including more established NBCs—than low- and middle-income countries (LMICs). Based on this assumption, the authors assumed that NBCs in these countries had remained relatively active during the pandemic, which would allow for the identification of discernible publication trends.

Three of the committees discussed in this study were not established nationally. The Commission de l’éthique en science et en technologie (Commission of Ethics of Science and Technology) in Canada was established with the advice of the government of Quebec; the Nuffield Council on Bioethics is an independent body in the United Kingdom; and the DH-BIO in the Council of Europe operates at an international level (see [1], Part 2, for NBCs at different levels of government). Nevertheless, given their comparable activities and long history, excluding these influential bodies would leave Canada, the UK, and Europe unrepresented, thereby creating a more significant gap in the comparative analysis. In particular, although CEST serves primarily in an advisory capacity to the Government of Québec and does not hold a national mandate, its inclusion does not distort the overall findings and helps provide a fuller picture of NBC-related activities during the COVID-19 pandemic.

### Collecting publications

The publications were sourced from their official webpages, referencing the World Health Organization’s “List of National Ethics Committees” [8]. Next, we categorized the collected documents based on the topics covered and publication date. This categorization was conducted during the period from July to September 2024.

The researchers collected articles that either (1) included terms such as “pandemic” or “COVID-19” in their titles, or (2) were published during the pandemic period and addressed moral concepts (e.g., equity) or the ethically relevant issues that had emerged during the pandemic (e.g., contact tracing applications). Articles that focused primarily on scientific or medical updates (e.g., vaccine development and distribution) or consisted of opinion columns were excluded from the analysis. This ensured that the analysis remained centered on the ethical considerations and contributions of NBCs during the pandemic.

Many NBCs published materials in languages other than English. As the authors are only proficient in Japanese, their native language, and English, the following approach was taken for classification. Step 1: Publications were categorized based on their titles. The titles of non-English and non-Japanese publications were translated using Google Chrome’s translation feature and ChatGPT, and the translations were compared to ensure accuracy. This step only applied to non-English materials. Step 2: the contents of all publications analyzed in this study were summarized in Japanese using ChatGPT using the prompt “Summarize the content of this publication in Japanese.” These summaries were then used to verify whether the earlier title-based classification was appropriate and accurate.

### Classifying the collected publications

The researchers investigated NBCs’ COVID-19 related publications, focusing on the temporal distribution and quantity of the information published. The following three aspects were analyzed: (1) total number of publications and country-specific publication counts, (2) trends in the number of publications every six months, and (3) classification of publications by content and trends in the number of publications over time for the top three categories. For aspect (3), the categorization was independently conducted by the authors and finalized through discussion and consensus. Preliminary testing revealed two main categories: issue analysis and thematic classification. Within the thematic categories, the publications were further classified according to their primary subject matter. Because no existing reference list was available for this topic, the authors developed the thematic framework based on the ethical issues and contexts addressed by each publication. The “primary subject” was determined by comparing the classifications made independently by two authors with the summaries generated by ChatGPT and identifying the topic most central to the publication based on its title and abstract.

## Results

### Number of committees and publications

**Fig. 1:**
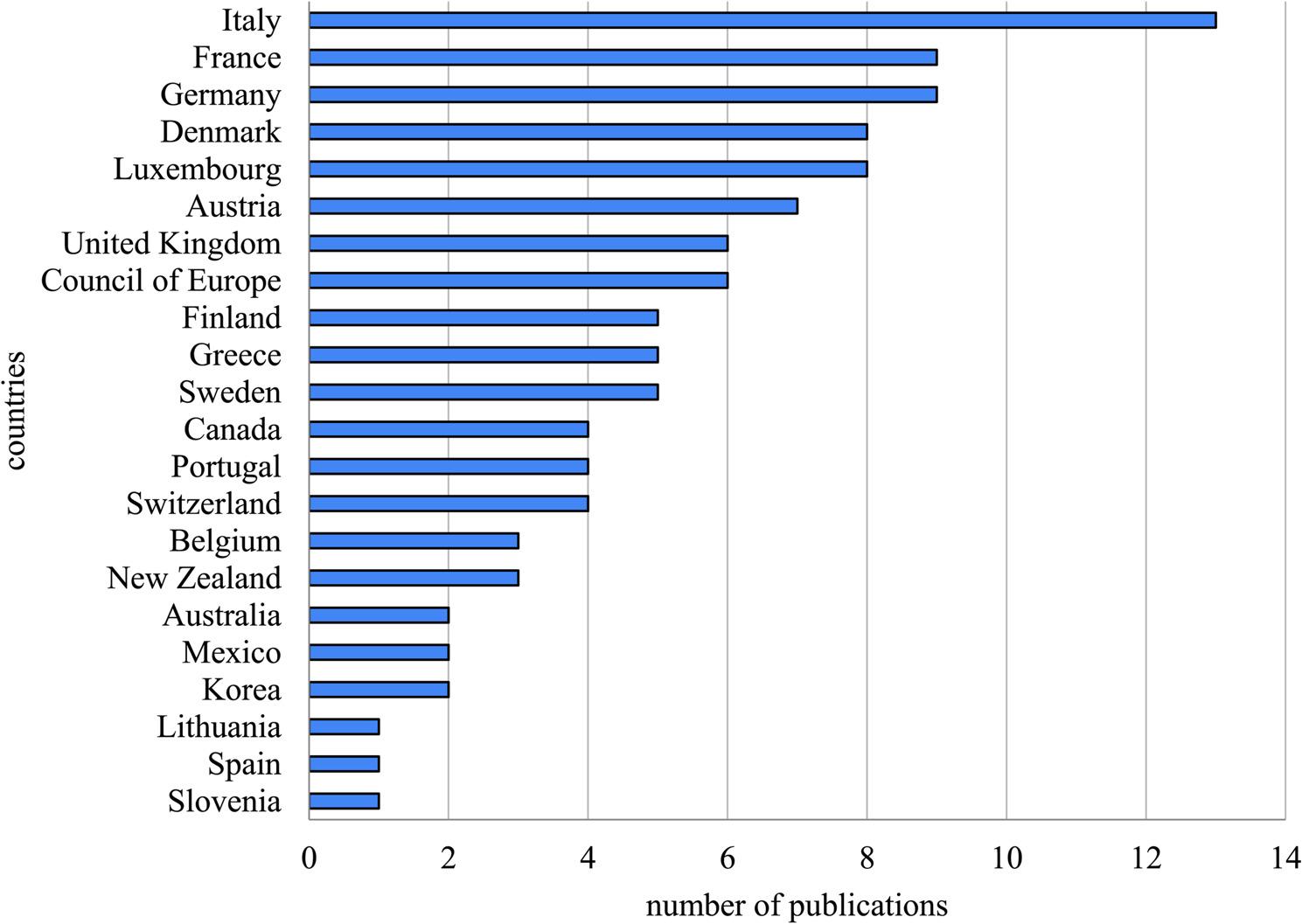
List of Countries/Organizations Arranged in Descending Order of Issued Statements. Note. Data were collected from the official websites and publications of NBCs in OECD member states, Singapore, Council of Europe. The countries and organizations are arranged in the descending order of statements issued

The study focused on 43 NBCs established in 35 countries and one organization. Among the OECD member countries, Chile, Colombia, and Costa Rica were excluded from the analysis because they were not included in the WHO’s list of National Bioethics Committees. Twenty-two (51%) committees out of the 43 investigated—located in 21 countries and one organization—published documents about ethical issues during the COVID-19 pandemic (Fig. 1; See the Appendix A for detailed results concerning all NBCs included in the analysis). These 22 NBCs issued 108 publications, with the highest number (13) coming from Italy’s Comitato Nazionale Per La Bioética (Italian Committee for Bioethics; Fig. 1).

### NBC publication patterns over time

**Fig. 2:**
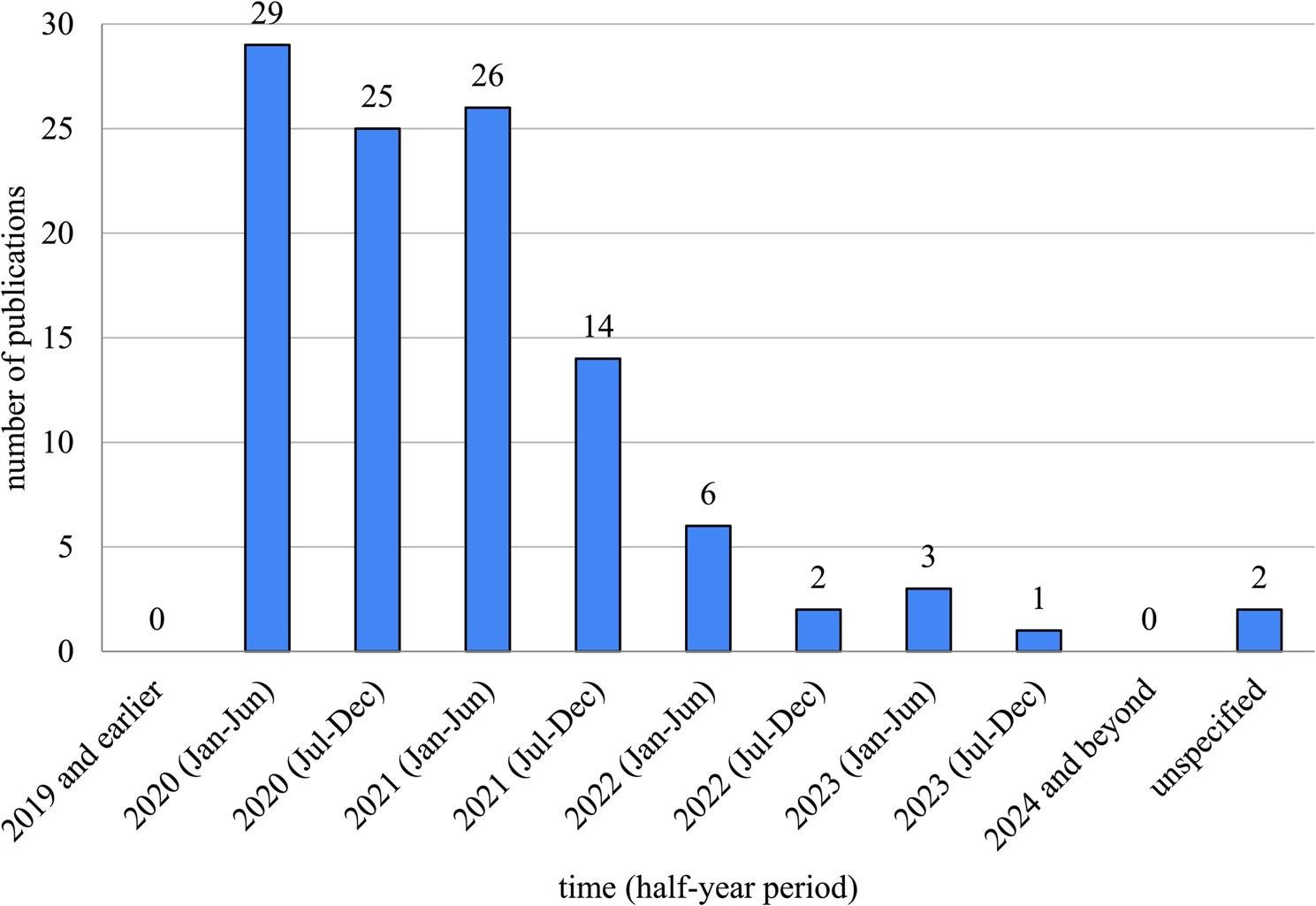
Semiannual Trends in the Number of Publications Issued by NBCs, 2019–2024. Note. Data were collected from official websites and publications of NBCs in OECD member countries, Singapore, Council of Europe. Each data point represents the total number of publications issued during each six-month period between 2019 and 2024

The NBCs’ publications were issued as follows: 54 in 2020, 40 in 2021, eight in 2022, and four in 2023 (two publications had unspecified publication dates) (Fig. 2). In addition, 32 out of 108 publications were issued within six months of the declaration of the public health emergency of international concern (PHEIC) on July 30, 2020 and 56 within one year.

### Themes Addressed in the Publications and Trends in the Timing

The publications were systematically categorized into two broad types and subsequently grouped under ten thematic categories overall. Type A publications aim to broadly organize a wide range of issues relevant at the time, while Type B publications focus on specific, concrete topics. Within each type, the themes are ranked in descending order of frequency, as shown in Table 1.

**Table 1:** Number of NBC Statements by Category

Type	Category/Subcategory	Number of Statements
Type A: General/Umbrella Statements	Early mapping of ethical issues	20
	How to respond to the next phase of COVID-19	1
Type B: Issue-Focused Statements	Vaccination (including vaccine distribution)	40
	* Mapping of the ethical issues around vaccination*	*(12)*
	* The ethical legitimacy of recommending vaccination to children and youths*	*(8)*
	* The need to prioritize vaccination for healthcare and long-term care workers*	*(5)*
	* Vaccination prioritization*	*(5)*
	* Differential treatment based on vaccination status*	*(3)*
	* The ethical legitimacy of mandatory vaccination*	*(2)*
	* Others*	*(5)*
	Resource allocation in clinical settings	13
	Tracing apps	9
	Vulnerable groups during COVID-19	7
	* Mapping of ethical issues around vulnerable groups*	*(3)*
	* Poor social contact of hospitalized patients*,* long-term care residents*	*(2)*
	* Impacts on children and youth (mainly due to poor social contact)*	*(2)*
	Ethics of clinical trials	6
	Vaccine passport / Green passport	5
	Lockdown	3
	Other	4

“Number of statements” refers to the total number of published statements within each thematic category identified during the study period. Subtopics (in italics) show more detailed subthemes mentioned within the main thematic category

**Fig. 3:**
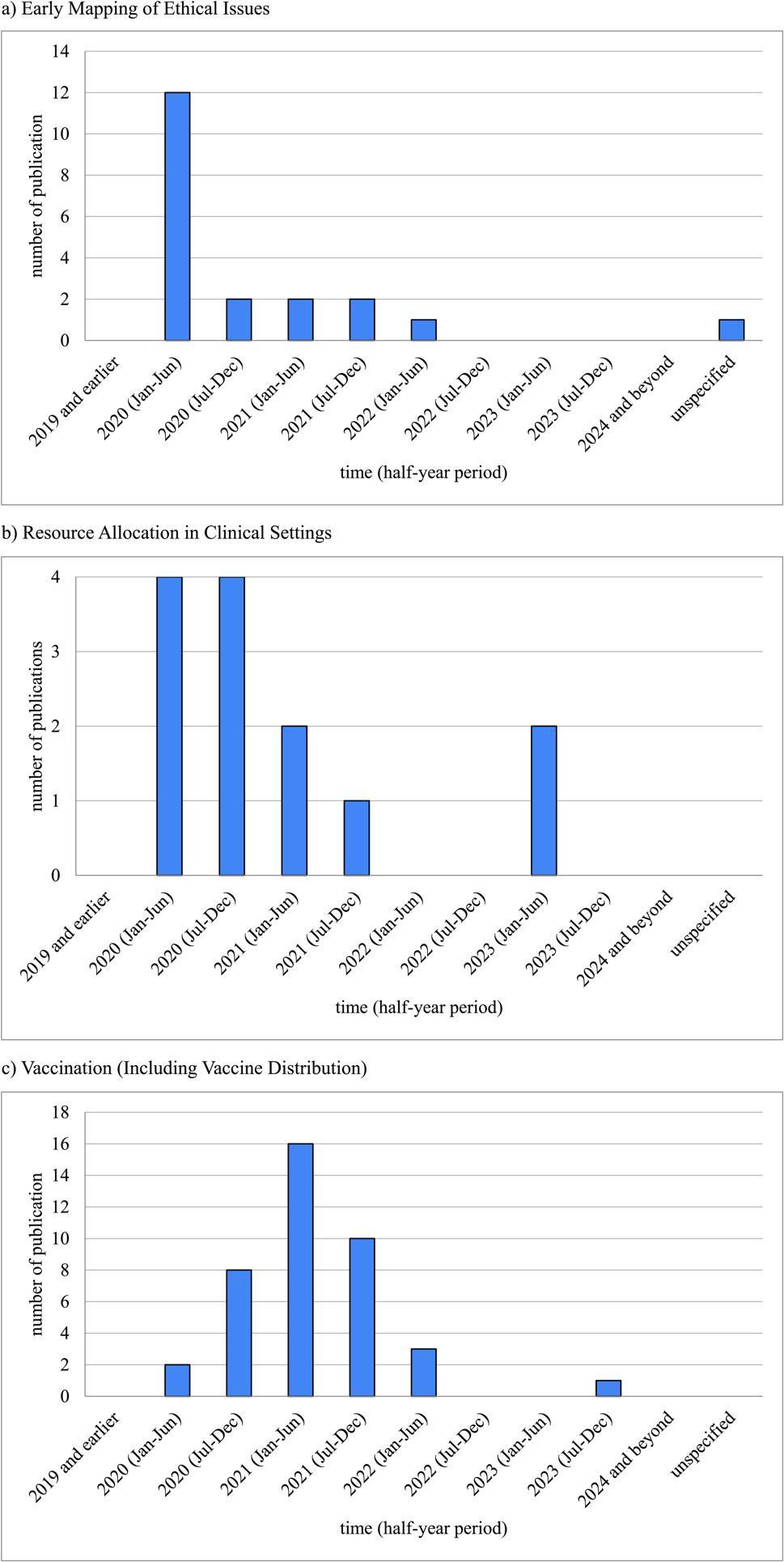
Semiannual Trends in the Top Three Publication Categories of NBCs, 2019–2024. Note. Data were collected from the official websites and publications of NBCs in OECD member countries, Singapore, and the Council of Europe. Each data point represents the total number of publications that mapped ethical issues related to COVID-19 and were issued during six-month periods between 2019 and 2024

The researchers hypothesized that for each of the 10 categories, there would be a discernible trend in the timing of the publication. For example, the three most frequently published categories identified in the previous section were “vaccination (including vaccine distribution),” “early mapping of ethical issues,” and “resource allocation in clinical settings” (Table 1). In total, 20 publications had content on the early mapping of ethical issues, of which 12 were published in the first half of 2020. The publications on resource allocation in clinical settings showed a similar trend, where eight of the 13 were issued in 2020: four publications in the first half and four in the second half of the year. The publications on vaccination (including vaccine distribution) first appeared in the first half of 2020 (*n* = 2), reached their peak in the first half of 2021 (*n* = 16), and then gradually declined over the following year (Fig. 3).

The researchers categorized the publications and their contents based on the month they first appeared to identify the observable trends. The first publications (*n* = 6) were issued in March 2020. The content of the six publications can be broadly categorized into two groups. Four publications were about early mapping of ethical issues in France, Germany, Greece, and the United Kingdom [9–12]. The other publications were on resource allocation in clinical settings in Austria and Luxemburg [13, 14].

## Discussion

This study analyzed the activities of NBCs during the COVID-19 pandemic by examining their publications. The findings indicate that NBCs surveyed in this paper were actively engaged, issuing publications promptly in response to key developments. Additionally, publications on vaccination aligned with global vaccine policy trends and peaked in the first half of 2021. Clinical trials for vaccines began in spring 2020, and approvals followed in late 2020, particularly in OECD and other Western nations. The European Union launched its vaccine rollout in December 2020. In addition to promptness, the publications covered a broad range of topics, indicating that they can serve as forums for ethical discussions during emergencies. This study also highlights the challenges NBCs face during urgent public health crises, such as the COVID-19 pandemic. Of particular importance are procedural issues, the NBC’s mission, and the NBCs’ limitations regarding international issues.

### Procedural issues

During the COVID-19 pandemic, NBCs addressed issues closely related to public health policies; however, these topics differed from the themes NBCs typically focus on. When discussing urgent public health crises, NBCs should also consider the following factors: (1) the transparency of the statement publication process, (2) the incorporation of statements into public policies, and (3) protections for those involved in drafting the statements. This section will discuss each in turn.

First, it has been proposed that there are five conditions for justifying public health policies, one of which is the requirement for public justification [7]. This condition requires public health agents to publicly explain and justify their policies. However, it remains unclear whether NBCs operated transparently amid the rapidly evolving circumstances of the COVID-19 pandemic, especially in the selection process for authors and the themes covered in these publications.

Second, while various infectious diseases have emerged in the past, the COVID-19 pandemic stands out in terms of its fast spread, global scale, prolonged duration, and deep impact on individual and societal behavior. Despite regional differences in timing and severity, its effects were felt worldwide. Although preparedness guidelines based on previous outbreaks (e.g., SARS, H1N1) were in place [15, 16], the recent move toward establishing the Pandemic Agreement suggests that those measures were insufficient for adequately addressing the challenges posed by COVID-19; consequently, policymakers may have consulted NBC publications when making decisions. It remains unclear whether these publications were incorporated into COVID-19 policies and, if so, to what extent they influenced policy decisions.

Third, the themes addressed in the NBC publications were all significant public health issues; however, some topics, such as medical resource allocation, were not only important but also particularly sensitive, as they involved decisions related to the prioritization of human lives. As a result, individuals who expressed opinions on such topics may have faced harsh criticism, defamation, or even demands to take responsibility for their views. Therefore, protecting publication authors is essential for NBCs to fulfill their advisory role during public health crises.

### Regional differences and divergent mandates of NBCs

The level of NBC activity differed across regions. Specifically, while NBCs in European countries actively issued statements, those in Asia were less active. Among the Asian countries examined in this study were South Korea, Japan, and Singapore. South Korea issued two publications, which focused on the ethical aspects of clinical trials [17, 18]. In contrast, the NBCs of Japan and Singapore did not issue any publications during the relevant period. This may be because the roles of NBCs in Japan and Singapore are specialized and concerned more with addressing ethical issues related to biotechnology than broader public health crises. These findings suggest that the roles assigned to NBCs differ across regions and countries.

### Absence of global ethical considerations

Only eight publications discussed cross-border ethical concerns during the COVID-19 pandemic, such as the equitable distribution of vaccines to low-income countries. Of these eight publications, only two were entirely devoted to international issues [19, 20]. As exemplified by the WHO’s slogan (“No one is safe until everyone is safe”) and the establishment of COVAX (COVID-19 Vaccines Global Access) to promote fair international vaccine distribution, global ethical considerations were highly significant during the COVID-19 pandemic [21].

### Limitations and future research directions

This study investigated the activities of NBCs that function as permanent bodies in various countries and regions. However, during the COVID-19 pandemic, temporary consultative bodies—such as government-led ad hoc committees and voluntary groups of researchers—may have played a significant role. It should be emphasized that this study did not intend to overlook such activities. While the focus here was on how permanent bioethics institutions operated during the pandemic, further research on these temporary bodies would likely shed additional light on the role that permanent organizations have played—or ought to play—in times of public health emergencies such as pandemics.

Moreover, this study did not examine the activities of NBCs in LMICs. The pandemic experience in these contexts differed significantly from that of high-income countries, particularly due to the shortages of medical resources and vaccines. Further research is needed to assess the extent of NBCs’ engagement in LMICs and to identify the specific ethical issues they addressed.

The severity and progression of the pandemic varied significantly across countries, and the social and economic impacts of COVID-19 depended on national contexts. Consequently, the timing of statements issued by national bioethics committees may not directly reflect the epidemiological situation in each country. However, data from sources such as the WHO suggest that—except for a few countries like Australia and New Zealand—the initial wave of the pandemic occurred at a relatively similar time in most countries [22]. While the extent of the pandemic’s impact differed, it is notable that many countries faced a simultaneous need to engage in public discussion and ethical deliberation in response to the unfolding crisis.

This study also identified three factors that NBCs should consider during the publication of their statements: (1) the transparency of the publication process, (2) the incorporation of statements into public policies, and (3) the protection of those involved in drafting the statements. As noted earlier, it remains unclear whether (and to what extent) NBCs followed these procedures during the pandemic. Therefore, a more detailed analysis of their publication practices is required to assess whether NBCs fulfilled their roles appropriately. This issue is essential for informing future pandemic preparedness.

Another limitation of this study was the use of ChatGPT for translation when analyzing non-English and non-Japanese publications. While AI-assisted translation inevitably involves some degree of uncertainty, the focus of the analysis was on classifying publications into thematic categories rather than conducting an in-depth analysis of complex argumentation or nuanced phrasing. Therefore, the researchers believe that any limitations in AI translation had minimal impact on the overall validity of the categorization process. Moreover, while methodological limitations prevented formal inter-coder reliability testing, there was a high degree of initial agreement between raters even though the first round of categorization was conducted independently. Thus, the categorization process proved to be largely straightforward. To enhance transparency and facilitate replication, a complete list of the publications analyzed is included in Appendix B, allowing readers to independently verify the classification decisions.

## Conclusions

This study investigated and analyzed trends in the publications issued by NBCs during the COVID-19 pandemic. These publications were released promptly in response to the evolving situation, and their content covered a wide range of topics. This study also identifies three challenges. First, further scrutiny is required to understand the processes preceding and following the publication of these documents. Second, significant regional disparities exist in NBC activity levels. Finally, the findings underscore the need for a more coordinated and globally inclusive approach to bioethical governance in future public health emergencies. This study relied solely on publicly available online information because public health-related information is generally open to the public. We believe that this study effectively captured most of the included NBCs’ outputs. Given the lack of similar investigations, this study serves as an important trend analysis in its field. 

## Data Availability

The data of this study are available upon reasonable request.

## Appendix

### Appendix A: List of committees examined in this study

**Table:**

Country/Organization	Commission Name	Publications
Italy	The National Bioethics Committee	13
France	The National Consultative Ethics Committee for Health and Life Sciences	9
Germany	The German Ethics Council	9
Denmark	Danish Council of Ethics	8
Luxembourg	Commission Consultative Nationale d’Ethique	8
Austria	The Bioethics Commission	7
Council of Europe	DH-BIO (The Committee on Bioethics)	6
United Kingdom, Northern Ireland	Nuffield Council on Bioethics	6
Finland	National Advisory Board on Social Welfare and Health Care	5
Greece	Hellenic National Bioethics Commission	5
Sweden	Swedish National Council on Medical Ethics	5
Canada	Commission de l’éthique en science et en technologie	4
Portugal	Conselho Nacional de Ética para as Ciências da Vida	4
Switzerland	Swiss National Advisory Commission on Biomedical Ethics	4
Belgium	Belgian Advisory Committee on Bioethics	3
New Zealand	National Ethics Advisory Committee	3
Australia	Australian Health Ethics Commission	2
South Korea	National Bioethics Committee	2
Mexico	Comisión Nacional de Bioética	2
Lithuania	Lithuanian Bioethics Committee	1
Slovenia	the Slovenian National Medical Ethics Committee	1
Spain	Comité de Bioética de España	1
Czech Republic	The Bioethics Commission of the Research and Development Council	0
Estonia	Estonian Council of Bioethics	0
Finland	National Advisory Board on Research Ethics	0
Iceland	National Bioethics Committee of Iceland	0
Japan	Bioethics Council Safety Committee for Science and Technology	0
Norway	The National Committees for Research Ethics	0
Singapore	Bioethics Advisory Committee	0
Switzerland	Federal Ethics Committee on Nonhuman Biotechnology	0
Canada	Canadian Institutes of Health Research	N/A^1^
Czech Republic	Central Ethics Committee of the Ministry of Health of the Czech Republic	N/A^2^
Denmark	The National Committee on Health Research Ethics	N/A^2^
Finland	National Advisory Board for Biotechnology	N/A^2^
Hungary	Egészségügyi Tudományos Tanács Tudományos és Kutatásetikai Bizottsága	N/A^2^
Ireland	Irish Council for Bioethics	N/A^3^
Israel	Bioethics Committee of the Israel Academy of Science and Humanities	N/A^2^
Latvia	Central Medical Ethics Committee	N/A^2^
Netherlands	The Health Council of the Netherlands	N/A^1^
Poland	Bioethics Commission, Ministry of Health	N/A^2^
Slovakia	Ethics Committee at the Ministry of Health of the Slovak Republic	N/A^2^
Türkiye	Bioethics Committee - UNESCO National Commission of Turkey	N/A^2^
United Kingdom, Northern Ireland	Central Office for Research Ethics Committees (COREC)	N/A^2^

1: Although it was listed in the “List of National Ethics Committees,” it functions not as a national bioethics committee but as a funding agency or a scientific advisory body

2: There was no website available

3: It ceased operations in 2010

### Appendix B: NBCs and publication titles

**Table:**

Country/ Organization	NBC	Publication title
Australia	Australian Health Ethics Committee	Decision-making for pandemics: An ethics framework
		COVID-19: Guidance on clinical trials for institutions, HRECs, researchers and sponsors
Austria	The Bioethics Commission	Management of scarce resources in healthcare in the context of the COVID-19 pandemic
		Vaccination against diseases for which there are approved vaccines in times of the COVID-19 pandemic
		Contact tracing in the COVID-19 pandemic
		Ethical questions about vaccination against COVID-19
		Legal and ethical issues in connection with people who have been vaccinated or who have recovered from infection during the COVID-19 pandemic
		Vaccination against COVID-19 as a prerequisite for practicing a medical or healthcare profession
		A pandemic is not a private matter
Belgium	Belgian Advisory Committee on Bioethics	On the ethical standards for the rollout of anti-COVID-19 vaccination for the benefit of the Belgian population
		Ethical aspects regarding prioritisation of care in times of Covid-19
		On the ethical legitimacy of prioritisation in healthcare
Canada	Commission de l’éthique de la science et de la technologie	Cadre de réflexion sur les enjeux éthiques liés à la pandémie de COVID-19
		Enjeux éthiques de la pandémie de COVID 19: précaution et déconfinement
		Les enjeux éthiques de l’utilisation d’une application mobile de traçage des contacts dans le cadre de la pandémie de COVID-19 au Québec
		Repères éthiques pour l’allocation équitable des médicaments prometteurs pour la covid-19 en contexte de rareté
Council of Europe	DH-BIO (The Committee on Bioethics)	Recommendation CM/Rec(2023)1 of the Committee of Ministers to member states on equitable access to medicinal products and medical equipment in a situation of shortage
		Statement on human rights considerations relevant to “vaccine pass” and similar documents
		Protection of human rights and the “vaccine pass”
		COVID-19 and vaccines: Ensuring equitable access to vaccination during the current and future pandemics
		A Council of Europe contribution to support member states in addressing healthcare issues in the context of the present public health crisis and beyond
		DH-BIO statement on human rights considerations relevant to the COVID-19 pandemic
Denmark	Danish Council of Ethics	COVID-19: Etik i en krisetid
		Etiske hensyn ved digital kontaktopsporing
		Etik og prioritering: COVID-19 og samfundsnedlukningens dilemmaer
		Etiske hensyn ved visitation og prioritering af patienter på ‘den røde kurve’
		COVID-19, sårbare og etik
		Balancen mellem frihed og sikkerhed
		COVID-19 og vaccination
		Høringssvar, frivillig tilvalgsordning for vaccination
Finland	National Advisory Board on Social Welfare and Health Care	Kannanotto Sosiaali- ja terveysalan eettiset periaatteet ovat voimassa myös poikkeusoloissa
		Lausunto Esityksestä kontaktien jäljityssovelluksen käyttöönotosta Covid-19-epidemian hallinnan tueksi
		Lausunto hallituksen esityksestä laiksi tartuntatautilain väliaikaisesta muuttamisesta
		Lausunto tartuntatautilain muutoksista
		Lausunto koronarokotteiden ensimmäisistä kohderyhmistä
France	The National Consultative Ethics Committee for Health and Life Sciences	La contribution du CCNE à la lutte contre COVID-19: Enjeux éthiques face à une pandémie
		Enjeux éthiques lors du dé-confinement: Responsabilité, solidarité et confiance
		Enjeux éthiques de la prise en charge et de l’accès aux soins pour tous en situation de forte tension liée à l’épidémie de Covid-19
		Enjeux éthiques soulevés par la vaccination contre la Covid-19
		Enjeux éthiques relatifs à la vaccination contre la Covid-19 des enfants et des adolescents
		Avis 137 Éthique et santé publique
		Réponse au Ministère des Solidarités et de la Santé sur les enjeux éthiques de la vaccination des enfants de 5 à 11ans contre la Covid-19
		Avis 140 « Repenser le système de soins sur un fondement éthique. Leçons de la crise sanitaire et hospitalière, diagnostic et perspectives »
		Avis 144 La vaccination des professionnels exerçant dans les secteurs sanitaires et médico-sociaux : Sécurité des patients, responsabilité des professionnels et contexte social
Germany	The German Ethics Council	SOLIDARITÄT UND VERANTWORTUNG IN DER CORONA-KRISE
		IMMUNITÄTSBESCHEINIGUNGEN IN DER COVID-19-PANDEMIE
		WIE SOLL DER ZUGANG ZU EINEM COVID-19-IMPFSTOFF GEREGELT WERDEN?
		MINDESTMASS AN SOZIALEN KONTAKTEN IN DER LANGZEITPFLEGE WÄHREND DER COVID-19-PANDEMIE
		BESONDERE REGELN FÜR GEIMPFTE?
		ZUR IMPFPFLICHT GEGEN COVID-19 FÜR MITARBEITENDE IN BESONDERER BERUFLICHER VERANTWORTUNG
		ETHISCHE ORIENTIERUNG ZUR FRAGE EINER ALLGEMEINEN GESETZLICHEN IMPFPFLICHT
		VULNERABILITÄT UND RESILIENZ IN DER KRISE – ETHISCHE KRITERIEN FÜR ENTSCHEIDUNGEN IN EINER PANDEMIE
		PANDEMIE UND PSYCHISCHE GESUNDHEIT. AUFMERKSAMKEIT, BEISTAND UND UNTERSTÜTZUNG FÜR KINDER, JUGENDLICHE UND JUNGE ERWACHSENE IN UND NACH GESELLSCHAFTLICHEN KRISEN
Greece	Hellenic National Bioethics Commission	Η Βιοηθική Διάσταση της Ατοµικής Ευθύνης στην Αντιµετώπιση του COVID 19 (κορωνοϊός)
		Σύσταση για την υποχρεωτικότητα του εµβολιασµού σε ορισµένες επαγγελµατικές οµάδες στον χώρο της υγείας
		Σύσταση για το ζήτηµα της προτεραιότητας στη διάθεση θεραπευτικών µέσων κατά του κορωνοϊού (µονοκλωνικά αντισώµατα)
		Κλινικές Δοκιµές εν Μέσω της Πανδηµίας COVID-19
		COVID-19 (Κορωνοϊός): Ζητήµατα Ιχνηλάτησης Κρουσµάτων και Επαφών τους
Italy	The National Bioethics Committee	COVID-19: LA DECISIONE CLINICA IN CONDIZIONI DI CARENZA DI RISORSE E IL CRITERIO DEL “TRIAGE IN EMERGENZA PANDEMICA”
		COVID-19: SALUTE PUBBLICA, LIBERTÀ INDIVIDUALE, SOLIDARIETÀ SOCIALE
		LA SPERIMENTAZIONE BIOMEDICA PER LA RICERCA DI NUOVI TRATTAMENTI TERAPEUTICI NELL’AMBITO DELLA PANDEMIA COVID-19: ASPETTI ETICI
		COVID-19 E BAMBINI: DALLA NASCITA ALL’ETÀ SCOLARE
		I VACCINI E COVID-19: ASPETTI ETICI PER LA RICERCA, IL COSTO E LA DISTRIBUZIONE
		LA SOLITUDINE DEI MALATI NELLE STRUTTURE SANITARIE IN TEMPI DI PANDEMIA
		URGENZA VACCINALE: ASPETTI BIOETICI
		PASSAPORTO, PATENTINO, GREEN PASS NELL’AMBITO DELLA PANDEMIA COVID-19 (PASS COVID-19): ASPETTI BIOETICI
		VACCINI E PLACEBO
		VACCINI ANTI-COVID-19 E ADOLESCENTI
		VACCINAZIONE ANTI-COVID-19 PER I BAMBINI DI 5/11 ANNI: RIFLESSIONI BIOETICHE
		LA COMUNICAZIONE ISTITUZIONALE NELL’AMBITO DELLA PANDEMIA: ASPETTI BIOETICI
		VACCINAZIONI ANTI COVID-19 E MIGRANTI
Lithuania	Lithuanian Bioethics Committee	Lietuvos bioetikos komiteto kreipimasis dėl atsakingo pacientų ir visuomenės informavimo apie vakcinaciją nuoCOVID-19
Luxembourg	Commission Consultative Nationale d’Ethique	Repères éthiques essentiels lors de l’orientation des patients dans un contexte de limitation des ressources thérapeutiques disponibles due à la crise pandémique du COVID-19
		Prise de position de la Commission Nationale d’Éthique (C.N.E.) sur les aides informatiques dans la lutte contre la pandémie du Coronavirus SARS-CoV-2
		Prise de position de la Commission Nationale d’Éthique (C.N.E.) sur la vulnérabilité de certaines personnes engendrée par la crise du COVID-19
		Avis sur les aspects éthiques relatifs à la priorisation des personnes à vacciner contre la COVID-19
		Avis consolidé sur les aspects éthiques relatifs à la priorisation des personnes à vacciner contre la Covid-19
		Avis sur la priorisation dans le cadre de la phase 6 de la stratégie vaccinale contre la Covid-19
		Réflexions sur la situation vaccinale des mineurs et une possible obligation vaccinale des majeurs dans le contexte de la pandémie de Covid-19
		Réponse de la Commission Nationale d’Éthique (C.N.E.) à la note de synthèse en vue du débat de consultation sur l’opportunité d’une obligation vaccinale dans le contexte de la pandémie de Covid-19
Mexico	Comisión Nacional de Bioética	La bioética ante la pandemia de COVID-19
		Bioética, vacunas y salud pública
New Zealand	National Ethics Advisory Committee	Ethics and equity: Resource allocation and COVID-19
		COVID-19 Vaccination status and access to health and disability research
		Public consultation: Ethical framework for resource allocation during times of scarcity
Portugal	Conselho Nacional de Ética para as Ciências da Vida	Tomada de Posição
		Deliberação Ética acerca da vacinação contra o Sars-Cov-2 de crianças entre os 5 e os 11 anos de idade
		Aplicações digitais móveis para controlo da transmissão da COVID-19
		Situação de emergência de saúde pública pela pandemia Covid-19: Aspetos éticos relevantes
Slovenia	National Medical Ethics Committee	Stališče komisije za medicinsko etiko Republike Slovenije o odločanju zdravnikov glede vključevanja respiratorjev v zdravljenje hudo prizadetih bolnikov zaradi COVID-19
South Korea	National Bioethics Committee	코비드19 관련 국가생명윤리심의위원장 성명_영문 (Statement on the COVID-19 pandemic from the chair of Korean National Bioethics Committee)
		면역글로블린 농축 치료제 개발을 위한 코로나바이러스감염증-19 완치자 혈장 채취 지침
Spain	Comité de Bioética de España	Declaración del comité de bioética de España sobre la estrategia de vacunación frente a la COVID-19 y, en especial, sobre priorización de la vacunación
Sweden	Swedish National Council on Medical Ethics	Etiska vägval vid en pandemi
		Uttalande: Smer ställer sig bakom WHO: s deklaration för en rättvis fördelning av vaccin
		Uttalande om vaccination mot covid-19 av vård- och omsorgspersonal
		Yttrande gällande etiska aspekter av ett erbjudande av vaccination mot covid-19 till 12–15 åringar i Sverige
		Yttrande vad gäller vaccination mot covid-19 av barn och unga mellan 5–11 år
Switzerland	Swiss National Advisory Commission on Biomedical Ethics	Die Covid-19-Impfung – Ethische Erwägungen zu Grundsatzfragen und spezifischen Anwendungsbereichen
		Policymaking regarding measures to control the SARS-CoV-2 pandemic: Ethical foundations
		Covid-19-Pandemie: Die gleichberechtigte Behandlung ungeimpfter Personen ist Pflicht
		Die COVID-19-Impfung bei Jugendlichen zwischen 12 und 15 Jahren. Ethische Erwägungen
United Kingdom, Northern Ireland	Nuffield Council on Bioethics	Ethics tools for decision-makers: Responding to public health threats
		Fair and equitable access to COVID-19 treatments and vaccines
		Ten questions on the next phase of the UK’s COVID-19 response
		Vaccine access and uptake
		COVID-19 antibody testing and “immunity certification”
		Ethical considerations in responding to the COVID-19 pandemic
